# Controlling the Spins Angular Momentum in Ferromagnets with Sequences of Picosecond Acoustic Pulses

**DOI:** 10.1038/srep08511

**Published:** 2015-02-17

**Authors:** Ji-Wan Kim, Mircea Vomir, Jean-Yves Bigot

**Affiliations:** 1 Institut de Physique et Chimie des Matériaux de Strasbourg, UMR 7504, CNRS, Université de Strasbourg, BP 43, 23 rue du Loess, 67034 Strasbourg Cedex 02, France

## Abstract

Controlling the angular momentum of spins with very short external perturbations is a key issue in modern magnetism. For example it allows manipulating the magnetization for recording purposes or for inducing high frequency spin torque oscillations. Towards that purpose it is essential to modify and control the angular momentum of the magnetization which precesses around the resultant effective magnetic field. That can be achieved with very short external magnetic field pulses or using intrinsically coupled magnetic structures, resulting in a transfer of spin torque. Here we show that using picosecond acoustic pulses is a versatile and efficient way of controlling the spin angular momentum in ferromagnets. Two or three acoustic pulses, generated by femtosecond laser pulses, allow suppressing or enhancing the magnetic precession at any arbitrary time by precisely controlling the delays and amplitudes of the optical pulses. A formal analogy with a two dimensional pendulum allows us explaining the complex trajectory of the magnetic vector perturbed by the acoustic pulses.

Since its discovery^1^, the ultrafast demagnetization and precession of the magnetization induced by femtosecond laser pulses has received intensive attention^2^,^3^,^4^,^5^,^6^,^7^,^8^,^9^,^10^,^11^,^12^,^13^,^14^. How fast and how efficiently spins can be controlled are crucial matters in the field of ultrafast magnetism. In that regards, a coherent control of the magnetization requires to impulse a sudden change of the spins angular momentum which results in a motion of precession of the magnetic vector around the effective field. This is generally achieved via a change of the material anisotropy of the considered magnetic system using femtosecond laser pulses as demonstrated in various configurations of pump and probe pulses designed for manipulating the spins in magnetic semiconductors^15^,^16^,^17^,^18^, dielectrics^19^,^20^ or metals^21^. The laser source can advantageously be a terahertz pulse^16^, a photo- or an optomagnetic pulse^20^. Recently we reported that magneto-acoustic pulses can also be used for modifying the magnetization vector in ferromagnetic materials^21^. Alternatively one can induce a spin torque transfer between magnetically coupled layers, for example in multilayered material structures, a process that is usually achieved with currents but which can also be optically manipulated^22^,^23^. Inducing a motion of precession is important for generating spin torque oscillations and being able to control them allows for example to selectively picking up single frequency modes in superimposed temporal oscillations^24^,^25^.

An important goal in controlling the angular momentum is not only to induce a torque at ultrashort times but also to be able to amplify or suppress the torque oscillations. It is the purpose of this work to show how to induce and manipulate at will the precession of the magnetization using a sequence of two or three acoustic pulses which are generated by femtosecond optical pulses. The ferromagnetic material is a nickel film but it can be any ferromagnetic or ferrimagnetic structure as long as the material has a large magnetostriction. Importantly, we study the precise conditions for such control by choosing the appropriate amplitudes and time delays between the pulses. The effect of the shapes of the acoustic pulses are also considered as, either unipolar or bipolar pulse can be generated via the lattice compression and expansion propagating in the magnetic material. To explain the effect of each particular sequence of acoustic pulses and the corresponding control of angular momentum, we make a formal analogy between the controlled motion of precession and a two dimensional pendulum subject to momentum kicks provided by the acoustic pulses. The trajectory of the magnetic vector results from both the change of the frequency of the corresponding pendulum and the amplitude of the torque. The model considers either unipolar and bipolar Crenel function pulses or realistic strain pulses. We first describe the control of the magnetization by two pulses and its pendulum analogy. Then we describe the case of three pulses, the pendulum analogy being further discussed in the supplementary information (SI). Finally we briefly discuss the effect of the pulse shapes which is also detailed in the (SI).

## Results and Discussion

### Experimental configuration and sample description

**Figure 1:**
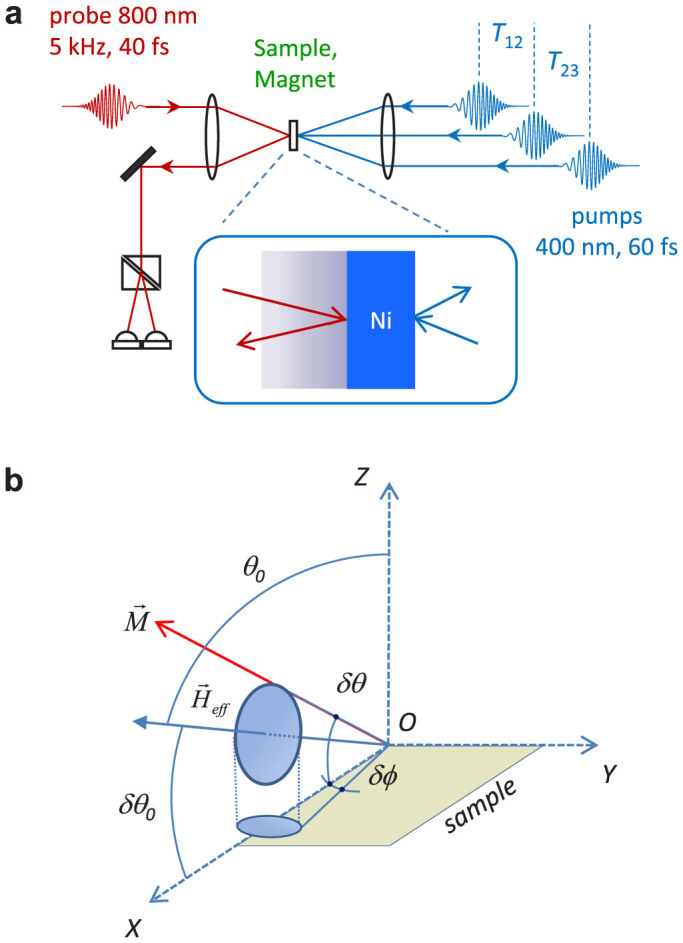
Time resolved magneto-acoustic experimental configuration. (a) Sketch of pump-probe magneto-acoustic set-up with backward probing. (b) Definition of Cartesian and spherical coordinates for the magnetization precession dynamics.

The experiment was performed by exciting the front side of Ni films using sequences of femtosecond pump pulses with controlled time delays and detecting the reflectivity and magnetization dynamics on the back side through a substrate using probe pulses, by means of the time-resolved pump-probe technique. Figure 1a is a sketch of the experimental configuration. The femtosecond pump pulses (60 fs, 400 nm) give rise to a thermal expansion of the lattice, which produces acoustic pulses in the front side of Ni. While propagating through the film, they bring about a modification of the magneto-crystalline anisotropy via a magnetostriction which takes place during the acoustic pulse, therefore initiating a precession of the magnetization vector. The probe pulses (40 fs, 800 nm) have an incident angle of 10° and measure both the transient differential reflectivity Δ*R*(*t*) and the differential magneto-optical polar Kerr rotation Δ*θ_K_*(*t*) on the back side as a function of the time delay *t* between a pump and probe. The signals are measured with a synchronous detection scheme^9^. We used a poly-crystalline 350-nm-thick Ni film deposited on a sapphire substrate by magnetron sputtering, which has the good acoustic impedance match for the purpose of the experiment (~10% of an acoustic pulse is reflected). The external magnetic field was chosen to be *H_ext_ = * 0.36 T with an angle *of* 44° with respect to the normal to the sample plane. In Fig. 1a *T*_12_ (respectively *T*_23_) represent the delays between pulses 1 and 2 (respectively 2 and 3). As shown hereafter, it is convenient to define the temporal quantities 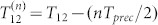 and 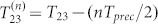, *T_prec_* being the period of the precession (74 ps in the following). It allows referring to a particular number of full rotations labeled by (*n*). We also define the total energy density *E_i_* of the *i*-th pump pulse as well as their ratio *β_ij_* = *E_i_*/*E_j_*(*i*, *j* = 1, 2, 3). Figure 1b sets the definition of spherical coordinates (*θ*, *ϕ*) and their corresponding small variations(*δθ*, *δϕ*) for the motion of precession as used hereafter, 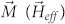 being respectively the magnetization vector and the effective field. The sample is assumed to be in the (*xOy*) plane and the magnetization initially points along (*θ*_0_ ≈ *π*/2, *ϕ* = 0) in the (*xOz*) plane.

### Control of the magnetization dynamics with a sequence of two acoustic pulses: amplification or suppression of the precession

#### Experimental data

**Figure 2:**
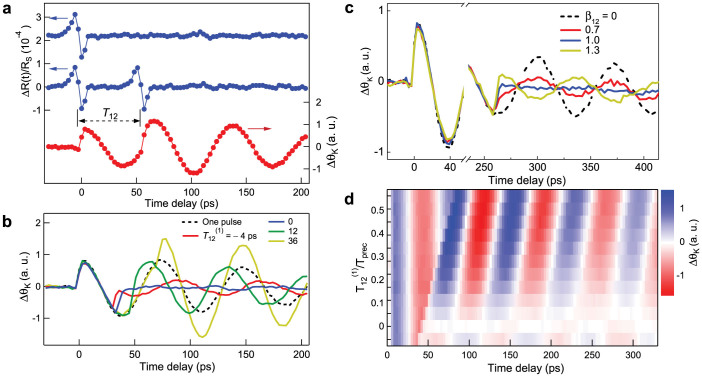
Control of magnetization dynamics with two acoustic pump pulses. (a) Differential reflectivity Δ*R/R_s_* with one and two pulses (top two curves) and differential Kerr signal Δ*θ_K_* for two pulses (lower curve) showing the timing sequence. (b) Δ*θ_K_* for various delays 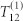 between pulses 1 and 2 with equal energy (*β*_12_ = 1). Dotted curve: reference signal obtained with one acoustic pump pulse. For 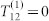 (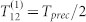): suppression (amplification) of precession. (c) Δ*θ_K_* for various *β*_12_ for the fixed delay 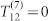. For *β*_12_ = 1: suppression of precession. (d) Two dimensional mapping of Δ*θ_K_* versus time *t* and 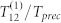. The suppression (or amplification) of precession occur for 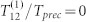 (or 1/2). The increment in 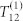 is 4 ps.

Let us first focus on a sequence of two independent acoustic pulses. The excitation pulse which is centered at *t* = 0 ps initiates the precession of the magnetization via magnetostriction and the control pulse, which arrives after at *T*_12_, modifies the trajectory of the precession which projection on the normal to the sample is observed via the rotation Δ*θ_K_*(*t*). The change of reflectivity Δ*R*(*t*) normalized to its static value *R_S_* is represented in Fig. 2a in the case of excitation by the pulse 1 only (upper curve), therefore showing the effect of the acoustic strain as detailed in Ref. 21. An example of a detailed sequence of two pump pulses is also shown both for Δ*R*(*t*) and Δ*θ_K_*(*t*). In Fig. 2b the Δ*θ_K_*(*t*) curves correspond to various delays *T*_12_ (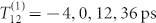). The dashed line, which corresponds to the case of only one pump pulse, serves as a temporal reference. Clearly the oscillations of the precession are suppressed for 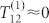 and are nearly doubled for 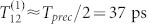. In Fig. 2c the Δ*θ_K_*(*t*) curves correspond to various amplitudes *β*_12_ (*β*_12_ = 0, 0.7, 1, 1.3) in the case of a fixed *T*_12_ = 7T*_prec_*/2 = 259 ps (note the broken scale in the temporal axis). A detailed view of the effect of varying 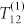 for *β*_12_ = 1 is provided in Fig. 2d by a two-dimensional mapping of the contrast of the oscillations as a function of time *t* and 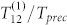 (still with *T_prec_* = 74 *ps*). Interestingly, the phase of the oscillations displays an abrupt change of π in the vicinity of 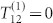 as seen by the opposite contrasts of colors for a fixed time *t* when 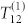 increases. As seen in Fig. 2c this abrupt change of π in the phase of the precession also occurs when *β*_12_ is varied across the value 1. For long delays like *T*_12_ = 7*T_prec_*/2 (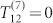) the precession is already significantly damped. Therefore, the value *β*_12_ = 1 has to be changed so that the motion of precession is exactly suppressed for 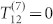. Summarizing the results, for a sequence of two pump pulses, the torque can be controlled such that the precession is suppressed for 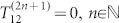 and amplified for 
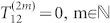
, β_12_ being finely adjusted near 1 to compensate for the damping.

#### Theoretical analysis

**Figure 3:**
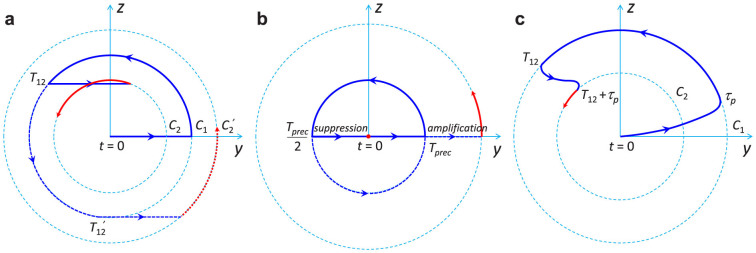
Schematic representation of controlled magnetization trajectory by two acoustic pulses, based on the pendulum analogy. (a) Trajectory corresponding to a decrease (full curve and circle *C*_2_) or an increase (dotted curve and circle C_2_') of the precession amplitude. (b) Trajectory corresponding to a full suppression (*T*_12_ = *T_prec_*/2) and maximum amplification (*T*_12_ = *T_prec_*) of the precession amplitude. (c) Trajectory showing the effect of pulse duration *τ_p_*.

Before considering a sequence of three pulses, let us analyze the preceding controlled behavior of the motion of precession in terms of amplitude and phase variations of the magnetic angular momentum. Towards that purpose we use the representation displayed in Fig. 3 where the precession of magnetization sketched in Fig. 1b is projected onto the (*yOz*) plane. As derived in section 1 of the (SI), the equations of motion for small angle deviations (*δθ*, *δϕ*) around the equilibrium (*θ*_0_, 0) are:
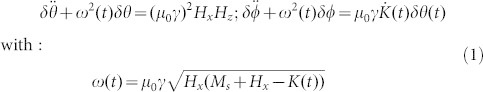
 *K*(*t*) = *K_az_*(*t*) + *K_sz_*(*t*) is an effective anisotropy corresponding to the magneto-crystalline *K_az_*(*t*) and strain *K_sz_*(*t*) anisotropies perturbed by the acoustic pulses, *M_s_*, *H_x_*, *H_z_* and γ are the magnetization at saturation, the *x* and *z* components of the external field and the gyromagnetic factor. Figure 3a shows the trajectory in the (*δθ*, *δϕ*) plane (equivalently (*yOz*)) due to a sequence of two delta-function pulses, starting from the equilibrium (center at *t* = 0). Because the first acoustic pulse modifies the anisotropy along *Oz*, angular momentum is acquired in δϕand the precession results on the circle *C*_1_, as indicated by the arrow, at a frequency 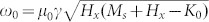. The radius of this circle corresponds to the amplitude of the precession about the static effective field 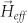, i.e. in equilibrium. For a given time delay *T*_12_, the second pulse abruptly modifies the trajectory of 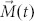 which continues its motion of precession with a smaller amplitude on the inner circle *C*_2_ and with a different phase. If the second acoustic pulse arrives at a later time 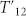, the trajectory evolves on the outer circle 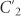 corresponding to a larger amplitude of the precession. As discussed in section 1 of the (SI), the real trajectories are more elliptical as the angular momentum goes to *δϕ*. In addition it slightly deviates from an ellipse because the tip of the magnetization evolves along the edges of a saddle shape. Two sequences of times are of particular interest as shown in Fig. 3b: the cases *T*_12_ = *T_prec_*/2 (or 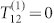) and *T*_12_ = *T_prec_* (or 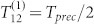) lead respectively to the suppression and to the maximum amplification of the motion of precession. Experimentally, they correspond to 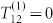 and 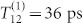 in Fig. 2b. They also show up in the experimental mapping displayed in Fig. 2d in the vicinity of 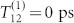 where the contrast changes abruptly and near 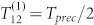 where the contrast is enhanced for a given time delay.

The preceding graphical representation can be easily carried on when the acoustic pulses have a finite duration *τ_p_*. As shown in Fig. 3c the trajectories also evolve along the *C*_1_ and *C*_2_ circles which are reached after an elapse of time. By making the analogy between equation (1) and the motion of a pendulum we show in section 1 of the (SI) that:
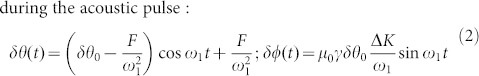

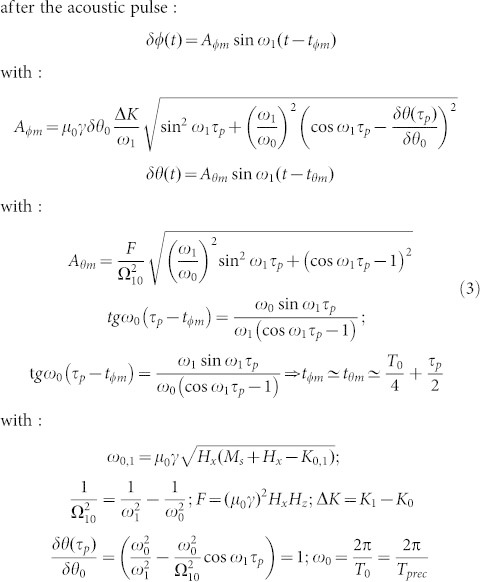
 This solution is obtained for an anisotropy *K*(*t*) which has a Crenel temporal shape of duration τ*_p_*with an amplitude 
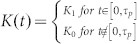
. As discussed in detail in section 1 of the (SI), the elapse time is equal to *τ_p_*, the particular precession amplitudes (*δθ* = *A*_*θ_m_*_ + *F*/ω_0_, *δϕ* = 0) occur for *T*_12_ = (*T_prec_*/4) + (*τ_p_*/2) and more importantly, with a second identical pulse the suppression of the motion of precession occurs for:
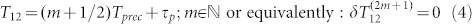
 The equality is obtained for delta-function pulses.

In the preceding analysis we have excluded any thermal effects since for thick metallic films, as the one used here (350 nm), the heat diffusion on the backside of the film leads to a slow exponential increase of the temperature in the time scale larger than 300 ps that is much larger than the precession period. Precisely this exponential temperature raise has been quantified to be ~10 K after ~400 ps in the case of 200 nm thick Ni film.

### Control of the magnetization dynamics with a sequence of three acoustic pulses: arbitrary choice of the timing for the precession control

#### Experimental data

**Figure 4:**
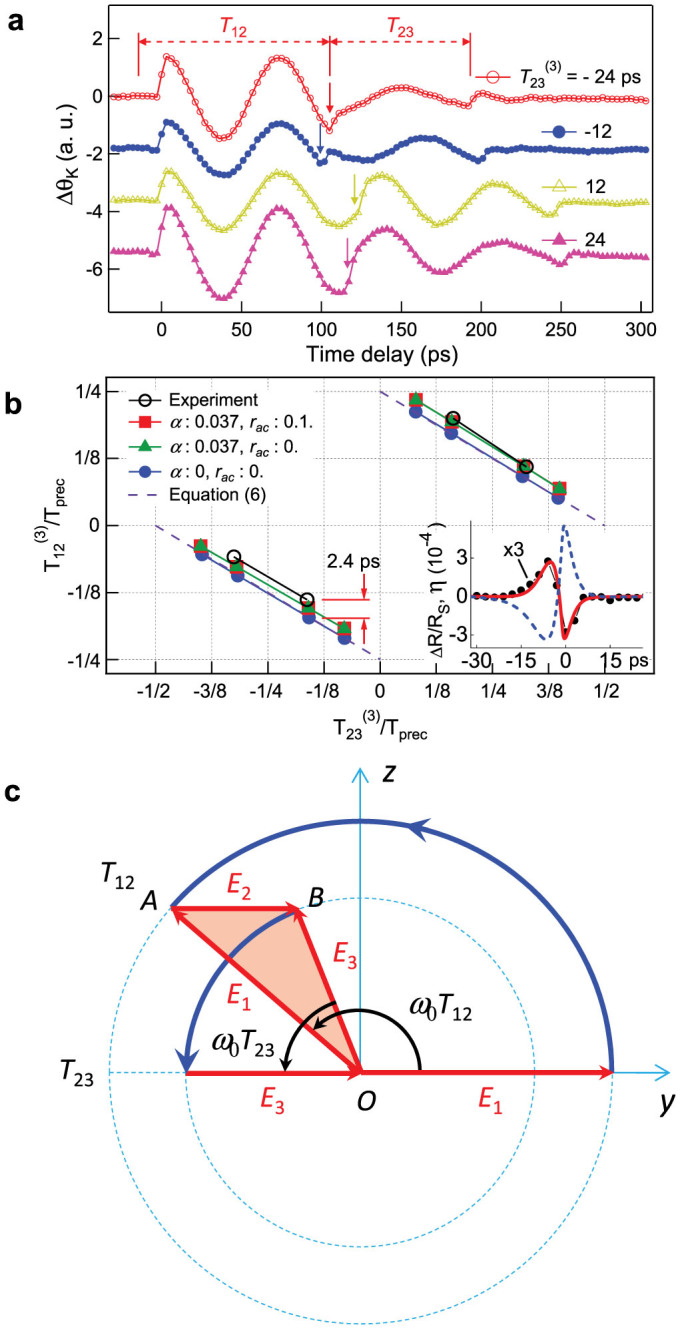
Control of magnetization dynamics with three acoustic pump pulses. (a) Δ*θ_K_* for various delays *T*_12_ and *T*_23_ (for *β*_23_ = 1). Fixing *T*_23_ arbitrarily *T*_12_ is adjusted to always suppress the precession. (b) Relation between 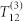 and 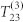
(for *β*_23_ = 1) for suppressing the precession and controlling the phase (advanced: upper curves; retarded: lower curves). Open circle: experimental results; rectangle, circle, and triangle: theoretical results for different conditions of Gilbert damping *α* and acoustic reflection coefficient *r_ac_*. Rectangles: (*α* = 0.037; *r_a_*_c_ = 0.1); triangles: (*α* = 0.037; *r_ac_* = 0); circles: (*α* = 0; *r_ac_* = 0; dashed lines: equation (6). Inset: effective strain pulse *η*(*t*) (dashed line), differential reflectivity (closed circles: measured; full line: calculated). (c) Schematic representation of controlled magnetization trajectory by three acoustic pulses leading to equation (6). The trajectory (full curve) is chosen to suppress the precession.

So far, we have employed two sequential acoustic pulses to control coherently the magnetization 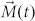. The constraint imposed by equation (4) is however restrictive as ultimately one would like to manipulate (stop or amplify) the precession at any time. In addition, in some cases acoustic pulses can have different shapes such as when generated from different generator transducers^26^ or pump lasers with different photon energies^27^. Also, other acoustic modes with different shapes can be produced with one pump pulse^28^,^29^. Moreover, the acoustic pulse can modify its shape during propagation as a result of phonon dispersion and nonlinearity^30^,^31^. Therefore, it is important to find the conditions for a complete control of the magnetization precession, independently of the pulse shape and independently of the particular precession frequency 2*π*/*T_prec_* of the ferromagnetic medium as imposed by equation (4). We achieved such control with a sequence of three pulses. In the following, three pulses are considered with respective time delays *T*_12_ and *T*_23_ which can be varied independently as well as the ratios *β_ij_*(*i*, *j* = 1, 2, 3). In Fig. 4a, the Δ*θ_K_*(*t*) curves correspond to various delays *T*_12_ and *T*_23_. The temporal sequence is chosen such that the motion of precession is well contrasted and distinct from the excitation pulse. For that purpose the delays *T*_12_ are varied near 3*T_prec_*/2, i.e. after one full revolution in the (*δθ*, *δϕ*) plane (equivalently (*yOz*)) has occurred. Therefore it is convenient to use the relative delays 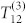 and 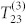 which is varied in a broad range (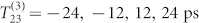) that covers more than half of a precession period (37 ps). Correspondingly, we search for the values 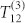 for which the precession is suppressed. In Fig. 4a, pulses 2 and 3 have the same amplitudes (*β*_23_ = 1), and the arrival time of the second pulse is indicated by the arrows. Clearly the constraint imposed by equation (4) is released. Instead, we can determine a relationship between the delays 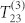 and 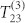 to control the precession. This is represented in Fig. 4b where we find that:
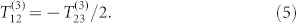


#### Theoretical analysis

The linear variation with slope −1/2 given by equation (5) allows setting the timing for the second and third acoustic pulses to suppress the precession of the magnetization at any time. It can also be deduced from the motion of a pendulum as derived in section 2 of the (SI). The jump corresponds to a phase shift which can be controlled to be negative (retarded phase lower curve) or positive (advanced phase in upper curve) by choosing appropriately the delay *T*_23_ between the pump pulses 2 and 3.

The constraint *β*_23_ = 1 is not necessary. The most general configuration that can be envisaged is represented in Fig. 4c. A sequence of pulses with different amplitudes and time delays is displayed so that at the end of pulse 3, the precession is suppressed (follow the trajectory). Considering the triangle OAB, the cosine and sine rules lead to:
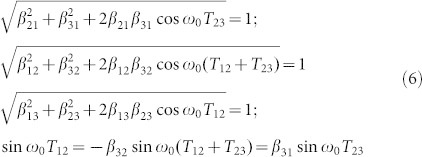
 Therefore one may arbitrarily choose a sequence of pulses and amplitudes to stop the precession providing that equation (6) is fulfilled. Two particular cases are interesting and summarized in equations (7): 1) the suppression of precession occurs after an advanced or a retarded phase shift (first column in equation (7)); 2) the amplification of precession occurs after an advanced or a retarded phase shift (second column in equation (7)). *E*_2_ = *E*_3_ (or *β*_23_ = 1) (the case of our experiment): 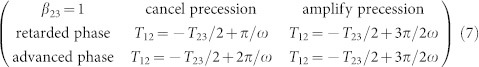
Let us now study the influence on the temporal delays *T*_12_ and *T*_23_ of additional effects like the presence of acoustic echoes reflected back and forth in the ferromagnetic film, as well as the damping of the precession. In particular, let us focus on the slight discrepancy at the origin in Fig. 4b (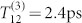 for 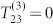). For that purpose, we performed simulations of the magnetization dynamics using the Landau-Lifshitz-Gilbert equation taking into consideration the magnetoelastic energy term: *E_me_* = −3/2*λ_s_σ_s_cos*^2^*θ* where *λ_s_* is the magnetostriction coefficient of a polycrystalline Ni film, *σ_s_* = 3*Bη*(1 − *ν*)/(1 + *ν*) the stress, *B* the Bulk modulus, *ν* the Poisson's ratio and η the strain profile. The modelling is further detailed in section 3 of the (SI). In the inset of Fig. 4b, the strain pulse *η*(*t*) (dashed line), defined as an effective quantity as reported in Ref. 21, is shown. It is obtained after fitting the transient reflectivity (solid line) from the experimental results Δ*R*(*t*)*/R_s_* (closed circle). The curve with squares in Fig. 4b is obtained for a Gilbert damping *α* = 0.037 obtained by fitting the experimental results. The acoustic echoes have been included with *r_ac_* = 0.1. For the triangles, the acoustic echoes are excluded (*α* = 0.037; *r_ac_* = 0) and for the closed circles the acoustic echo and the Gilbert damping are both ignored (*α* = 0; *r_ac_* = 0). From these graphs, it is clear that the offset can be partly attributed to the Gilbert damping which results in an offset of ~1.3 ps.

**Figure 5:**
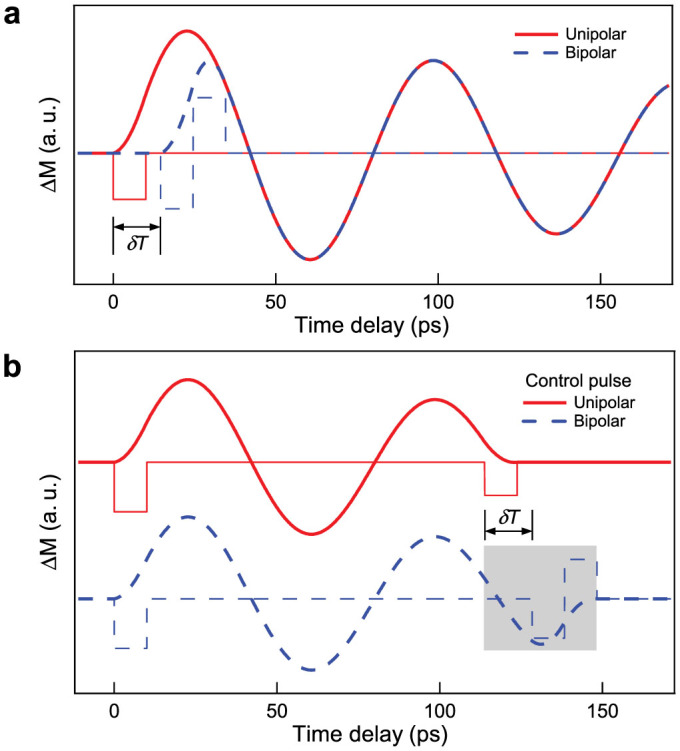
Control of magnetization precession with unipolar and bipolar pulses. (a) Modelling of the precession of magnetization induced by unipolar (full curve) and bipolar (dashed curve) pulses separated by the delay *δT*. (b) Coherent control of the magnetization precession with sequences of: two unipolar pulses (top full curve) and unipolar + bipolar pulses (lower dashed curve). Only the delays and amplitudes are important for the control (not the shape).

The preceding study shows that the control of the magnetization dynamics with acoustic pulses depends on the two delays 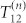 and 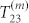 which values allow determining for example the cancellation of the precession as shown in Fig. 4b or its amplification. Let us stress that the particular individual shape of the acoustic pulses is not relevant as long as they are shorter than *T_prec_*. Only their relative delays and amplitudes can serve the purpose of controlling the magnetization vector via the change of the magneto-elastic anisotropy. To precise this concept, which is of major importance for applications, we have studied the effect of unipolar and bipolar shaped acoustic pulses on the magnetization trajectory. Figure 5a shows curves simulating the precession dynamics of magnetization induced by differently shaped acoustic pulses. The solid line corresponds to the precession of magnetization triggered by a 10 ps-long unipolar square pulse and the dashed line by a 20 ps-long bipolar squared one. The temporal delay *δT* between the two pulses is adjusted such that the two motions of precession are equal. When two pulses are used (dashed lines in Fig. 5b) clearly only the delay *δT* is important for the coherent control of the magnetization and not the detailed pulse shape as highlighted in the grey square. The first curve (full line) results from two unipolar pulses. The second curve (dashed one) corresponds to a unipolar excitation and bipolar control pulse. Only the delay *δT* determines when the suppression of the precession occurs. Overall, the conditions of coherent control can be determined by choosing the delay such that: *T* = *mT_prec_* + *δT* for the amplification and *T* = (*m* + 1/2)*T_prec_* + *δT* for the suppression of the precession of magnetization.

## Conclusion

We show that a sequence of two or three acoustic pulses is well adapted for controlling the spin torques in ferromagnetic materials, resulting either in the suppression or the amplification of the magnetization precession. When using two acoustic pulses, the control is somehow more restrictive because the occurrence of suppression or amplification is directly related to integers of half (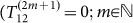) or full precession periods (
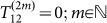
), quantities that strongly depend on the intrinsic material properties. However, in the case of three acoustic pulses, arbitrary delays *T*_12_, *T*_23_ and amplitudes *E_i_*(*i* = 1,2,3) can be used providing that equation (6) is satisfied. The second and third pulses then act as a single pulse which shape can be modified at will. A simple graphical representation of the magnetization trajectory follows. This picture is validated in the framework of the motion of a two dimensional pendulum subject to external pulsed perturbations. A full Landau-Lifshitz-Gilbert modelling of the dynamics, including the time dependent anisotropy resulting from the magneto-elastic changes induced by the acoustic strain, also provides the detailed influence of the material properties such as the precession damping. The consequences of this work are important whenever a precise control of the magnetization dynamics is desired. This is the case for example in spintronics for controlling spin torques but it can also be used for fundamental studies related to the lattice dynamics of materials particular when several individual modes co-exist. Let us finely emphasize that even though the duration of acoustic pulses is in the terahertz range, the control of the magnetization dynamics can be performed with an extreme precision as it is related to the time delays of the acoustic pulses, themselves generated by femtosecond optical pulses.

## Author Contributions

J.-W.K. did the experiments, prepared figures 2 to 5 and SI-2, did the numerical simulations for figure SI-2 and participated to the writing of the main manuscript. M.V. participated to the experiments. J.-Y.B. did the modelling and wrote the main manuscript and the supplementary information and did figures 1, 4 and SI-1. All authors reviewed the manuscript.

## Supplementary Material

Supplementary Information Supplementary Information
