# Supplementary material for: Controlling the Spins Angular Momentum in Ferromagnets with Sequences of Picosecond Acoustic Pulses
Source: Sci Rep. 2015 Feb 17;5:8511. doi: 10.1038/srep08511 (PMC4330532; doi:10.1038/srep08511)
Supplement: Supplementary Information [file srep08511-s1.pdf]

Controlling the Spins Angular Momentum in Ferromagnets with Sequences of Picosecond Acoustic Pulses

Ji-Wan Kim, Mircea Vomir, and Jean-Yves Bigot*

Institut de Physique et Chimie des Matériaux de Strasbourg, UMR 7504, CNRS, Université de Strasbourg, BP 43, 23 rue du Loess, 67034 Strasbourg Cedex 02, France.

Supplementary Information

This supplementary information is divided in two sections. In section 1 we make the analogy between the magnetization dynamics excited by acoustic pulses and a two dimensional pendulum by considering the Landau-Lifshitz equation in spherical coordinates $(\rho,\ \theta,\ \phi)$, in the approximation of a small deviation of the angles. The solution for a Crenel function perturbation pulse of the anisotropy is obtained. It allows making the connection between the dynamical measurements of the Kerr magneto-optical signal during and after the excitation of the Ni film by the first acoustic pulse, as described in the main text. In section 2 we consider the full dynamical model representing the dynamics of the magnetization precession using the Landau-Lifshitz-Gilbert equation, coupled to the dynamical change of the material anisotropy, taking into account the time dependent strain induced by the acoustic pulses.

Section 1. Precession of the magnetization in spherical coordinates with a time dependent anisotropy: analogy with a pendulum experiencing a fast perturbation of its angular momentum.

We consider the Landau-Lifshitz equation for the magnetization vector $\vec{M}$ of a ferromagnetic film, with constant modulus M_S, in spherical coordinates $\left(\rho = \left|\vec{M}\right| = M_S,\ \theta(t),\ \phi(t)\right)$, where θ and ϕ are the angles of $\vec{M}$ along the Oz axis and its projection onto the xOy plane along the Ox axis[1].

$$\begin{aligned} &\frac{d\vec{M}}{dt} = -\mu_0 \gamma \vec{M}(t) \times \vec{H}_{eff}(t) \\ &\frac{d}{dt}\frac{\partial L}{\partial \dot{q}_i} - \frac{\partial L}{\partial q_i} = 0 \qquad q_i = \theta,\ \phi \end{aligned} \tag{1.1}$$

γ is the gyromagnetic factor and μ_0 the magnetic permeability of vacuum. The equations of motion are given by the Lagrangian in equation (1.1) leading to:

$$\begin{aligned}\frac{d\theta}{dt} &= -\frac{\gamma}{M_s \sin\theta}\frac{\partial E}{\partial \phi}\\ \frac{d\phi}{dt} &= \frac{\gamma}{M_s \sin\theta}\frac{\partial E}{\partial \theta}\end{aligned} \tag{1.2}$$

$$E(t) = \left(-K_z(t) + \frac{\mu_0 M_S^2}{2}\right)\cos^2\theta(t) - \mu_0 M_S H_z \cos\theta(t) - \mu_0 M_S H_x \cos\phi(t)\sin\theta(t) \tag{1.3}$$

Without loss of generality we consider the sample to be in the *xOy* plane (see Fig. 1b of the main text), the anisotropy $\vec{K}$ along *Oz* and the external magnetic field $\vec{H}$ in the *xOz* plane. The free energy *E* contains three main contributions: the magnetocrystalline and magnetoelastic anisotropies K_z, the demagnetization energy due to the shape anisotropy ($\mu_0 M_S^2 \cos^2\theta / 2$) (tensor along *Oz* for the film) and the dipolar interaction $\vec{M}\cdot\vec{H}$. The perturbation by the acoustic pulses is taken care of by the effective variable $K_z(t)$ which contains both the change of magneto-elastic and crystalline anisotropy coefficient whenever it is relevant (see section 2 hereafter).

Equation (1.3) allows expressing (1.2) as:

$$\begin{aligned}\dot{\theta} &= -\mu_0\gamma H_x \sin\phi\\ \dot{\phi} &= \mu_0\gamma\left(\frac{2K_z}{\mu_0 M_S} - M_S\right)\cos\theta + \mu_0\gamma H_z - \mu_0\gamma H_x \frac{\cos\phi}{tg\theta}\end{aligned} \tag{1.4}$$

Where the dots denote the derivative with respect to time.

The perturbation by the acoustic pulses being small as sketched in Fig. 1b of the main text, we consider small angle deviations: $\theta = \frac{\pi}{2} - \delta\theta$ and $\phi = \delta\phi$, simplifying equations (1.4) :

$$\begin{aligned}&\delta\ddot{\theta} + \omega^2(t)\delta\theta = (\mu_0\gamma)^2 H_x H_z\\ &\delta\ddot{\phi} + \omega^2(t)\delta\phi = \mu_0\gamma\dot{K}(t)\delta\theta(t)\\ &\text{with: } \omega(t) = \mu_0\gamma\sqrt{H_x(M_s + H_x - K(t))}\end{aligned} \tag{1.5}$$

To simplify the notation, in the following we rewrite: $\delta\theta \to \theta$ and $\delta\phi \to \phi$, the initial equilibrium condition being: ($\delta\theta_0 \to \theta_0$; $\delta\phi_0 \to \phi_0 = 0$). In equations (1.5) the frequency $\omega(t)$ is introduced to put forward the similarity of the magnetization dynamics to the one of a pendulum when we consider Crenel or delta functions for the excitation by the acoustic pulses. Assuming

such a Crenel function which results in a change of the anisotropy between K_0 and K_1 during the excitation pulse of duration τ_p, the solutions of equations (1.5) are given by:

$$\begin{aligned} \theta(t) &= \theta_m \cos\omega_0(t - t_{m\theta}) + \frac{F}{\omega_0^2} \\ \phi(t) &= \phi_m \sin\omega_1(t - t_{m\phi}) \end{aligned} \tag{1.6}$$

During the excitation pulse:

$$\begin{aligned} &\theta(t) = F\left(\frac{1}{\omega_1^2} - \frac{1}{\Omega_{10}^2}\cos\omega_1 t\right) \\ &\phi(t) = \mu_0\gamma\theta_0\frac{\Delta K}{\omega_1}\sin\omega_1 t \\ &\text{with: } \omega_{0,1} = \mu_0\gamma\sqrt{H_x(M_s + H_x - K_{0,1})} \;\; ; \;\; \frac{1}{\Omega_{10}^2} = \frac{1}{\omega_1^2} - \frac{1}{\omega_0^2} \\ &F = (\mu_0\gamma)^2 H_x H_z \;\; ; \;\; \Delta K = K_1 - K_0 \;\; ; \;\; \theta_0 = \frac{(\mu_0\gamma)^2 H_x H_z}{\omega_0^2} = \frac{F}{\omega_0^2} \end{aligned} \tag{1.7}$$

After the excitation pulse:

$$\begin{aligned} &A_{\phi m} = \mu_0\gamma\theta_0\frac{\Delta K}{\omega_1}\sqrt{\sin^2\omega_1\tau_p + \left(\frac{\omega_1}{\omega_0}\right)^2\left(\cos\omega_1\tau_p - \frac{\theta(\tau_p)}{\theta_0}\right)^2} \;\; ; \text{ with: } \frac{\delta\theta(\tau_p)}{\delta\theta_0} = \left(\frac{\omega_0^2}{\omega_1^2} - \frac{\omega_0^2}{\Omega_{10}^2}\cos\omega_1\tau_p\right) = 1 \\ &tg\omega_0\left(\tau_p - t_{\phi m}\right) = \frac{\omega_0\sin\omega_1\tau_p}{\omega_1\left(\cos\omega_1\tau_p - 1\right)} \end{aligned} \tag{1.8}$$

$$\begin{aligned} A_{\theta m} &= \frac{F}{\Omega_{10}^2}\sqrt{\left(\frac{\omega_1}{\omega_0}\right)^2\sin^2\omega_1\tau_p + \left(\cos\omega_1\tau_p - 1\right)^2} \\ \text{tg}\omega_0\left(\tau_p - t_{\theta m}\right) &= \frac{\omega_1\sin\omega_1\tau_p}{\omega_0\left(\cos\omega_1\tau_p - 1\right)} \end{aligned} \tag{1.9}$$

Figure S1a shows the magnetization trajectory in the plane $(\theta(t),\ \phi(t))$. The values of the different parameters are:

$$\begin{aligned} &H = 2.5\times10^5\ \text{Am}^{-1};\ K_0 = 2.1\times10^4\ \text{Am}^{-1};\ \gamma = 1.76\times10^{11}\ \text{rad s}^{-1}\text{T}^{-1}; \\ &\mu_0 = 4\pi\times10^{-7}\ \text{Hm}^{-1};\ M_S = 5\times10^5\ \text{Am}^{-1};\ \Delta K = 0.5\times K_0;\ \tau_p = 5 \text{ and } 10\text{ ps} \end{aligned} \tag{1.10}$$

Note that the units of the effective anisotropies K_0 and ΔK are the ones of a field using: $K = \frac{k_a}{2\mu_0 M_S}$, k_a being time dependent anisotropy coefficients (magnetocrystalline and magnetoelastic).

The amplitudes θ_m, ϕ_m represent the values of $\theta(t)$, $\phi(t)$ at the times $t_{m\theta}$ and $t_{m\phi}$ when the tip of the magnetization vector reaches the top of the circle in Fig. S1a, therefore representing the motion in quadrature with respect to t=0. Two different trajectories are represented for the pulse durations $\tau_p = 5$ ps (dotted line) and $\tau_p = 10$ ps (full line) to show the initial inertia along the *Oy* direction (angle ϕ). Since the amplitude of the acoustic pulse is maintained constant (only its duration changes), the "pendulum" receives more total angular momentum for $\tau_p = 10$ ps. Even though the two trajectories are self-similar during the time τ_p the circular trajectory is reached earlier with the shorter pulse, but with less amplitude. Figure S1b shows the effect of a larger anisotropy with the same pulse duration $\tau_p = 10$ ps and $K_0 = 2.1\times10^4$ Am^{-1} with $\Delta K / K_0 = 0.1$ (dotted line) and $\Delta K / K_0 = 0.5$ (full line). Figures S1c and S1d show the variation of $t_{m\theta}$ (dashed line) and $t_{m\phi}$ (dotted line) as a function of the pulse duration for two cases of static anisotropies $K_0 = 2.1\times10^4$ Am^{-1} (Fig. S1c) and $K_0 = 2.1\times10^5$ Am^{-1} (Fig. S1d); in both cases $\Delta K / K_0 = 0.2$. The solid line represents the linear behavior $t_{m0} = (T_0/4) + (\tau_p/2)$ which corresponds to the case of a rigid pendulum as discussed in the next paragraph. For the lowest anisotropy the two times are nearly the same and they become different for larger anisotropies, inducing an elliptical trajectory with rotated axis. This is a consequence of the conservation of angular momentum which is equal to the temporal change of angular velocity. Therefore more momentum is transferred along the *Oy* axis (initial change of ϕ is faster). Note that in both cases the reference curve t_{m0} has a different offset as ω_0 depends on the static anisotropy.

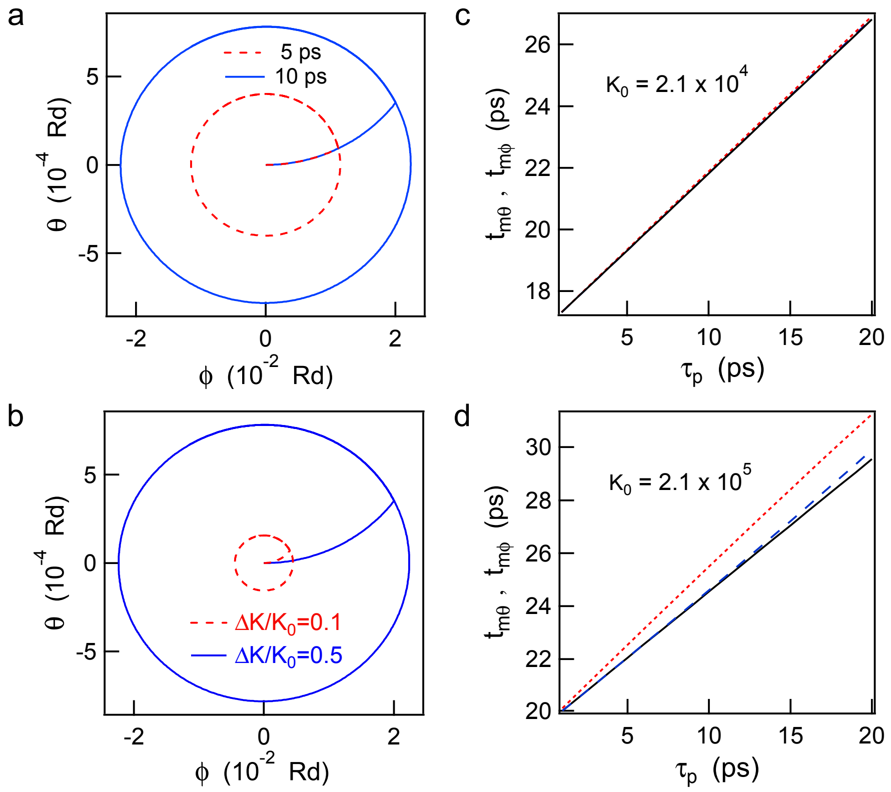

Figure S1: Trajectory of the magnetization excited by a Crenel function pulse. (a) Trajectories for two pulse durations τ_p=10 ps (full line) and τ_p=5 ps (dotted line). (b) Trajectories for two relative change of anisotropies $\Delta K/K_0$=0.5 (full line) and $\Delta K/K_0$=0.1 (dotted line) and for both: τ_p=10 ps , $K_0=2.1\text{x}10^4\ A\cdot m^{-1}$. (c) Variation of the time delays $t_{m\theta}$ (dashed line) and $t_{m\phi}$ (dotted line) as a function of the pulse duration τ_p for $K_0=2.1\text{x}10^4\ A\cdot m^{-1}$, $\Delta K/K_0$=0.2. (d) Same as (c) but $K_0=2.1\text{x}10^5\ A\cdot m^{-1}$.

Section 2. Description of the control of magnetization with acoustic pulses using the Landau-Lifshitz-Gilbert equation and the time dependent magneto-elastic anisotropy of nickel.

Including the damping and real temporal shapes for the acoustic pulses prevents from obtaining analytical solutions as done in the preceding section. To analyze the effect of such parameters as the damping α the magneto-elastic coefficients as well as the acoustic pulse shapes due to the material strain, we solve numerically the Landau-Lifshitz-Gilbert equation. Importantly our numerical simulations show that the particular pulse shape or damping does not affect the main characteristics of the control by the acoustic pulses. To show this statement, we have considered several cases of pulses as shown in Fig. S2. The modelling of the strain is performed like in our preceding work[2]. Figure S2a shows the magnetization trajectory in the

plane yOz for two Crenel unipolar pulses with different durations: $\tau_p = 5$ ps inner trajectory in dotted line and $\tau_p = 10$ ps outer trajectory in full line. As expected the behavior is the same as in the analytical case (Fig. S1a). For a bipolar pulse with compressive strain duration τ_p^{comp} and tensile duration τ_p^{tens} we obtain the trajectories shown in Fig. S2b. The inner circle corresponds to: $\tau_p^{comp} = \tau_p^{tens} = 5$ ps and the outer one to: $\tau_p^{comp} = \tau_p^{tens} = 10$ ps. The trajectories are affected only during the pulse with first an excursion along Oy ($+\phi$) due to the corresponding positive angular momentum and during the tensile part the excursion tends to go along -Oy ($-\phi$). But the equal amplitudes of the compressive and tensile parts of the strain do not bring the magnetization back to equilibrium because meanwhile it has acquired momentum also along Oz (θ angle). Finally, using the temporal shape of the effective strain displayed in Fig. S2c leads to the trajectory shown in Fig. S2d. The behavior is similar to the bipolar Crenel except for the rounder approach to well-defined circular trajectories.

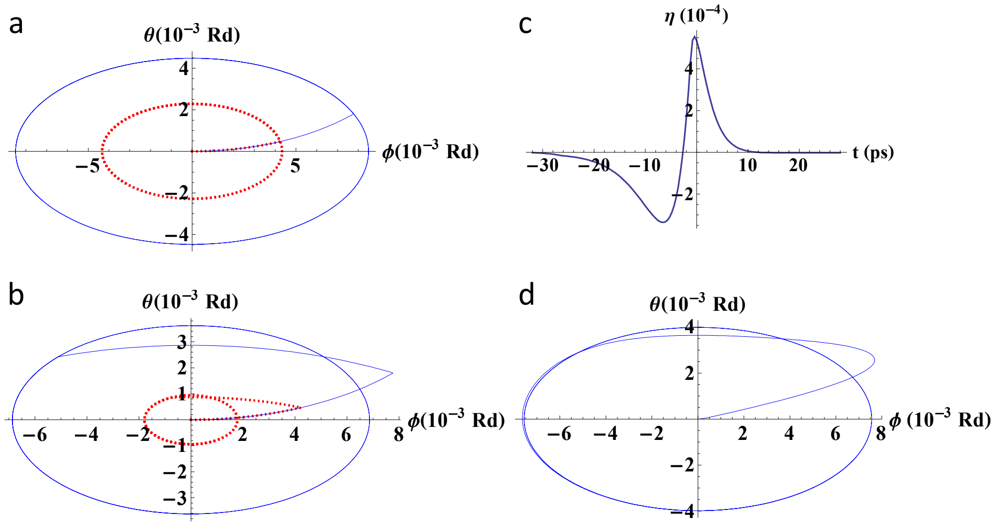

Figure S2: Model of trajectory of the magnetization in nickel film using the full LLG model with a time dependent strain. (a) Trajectories for unipolar Crenel function pulses with durations τ_p=10 ps (full line) and τ_p=5 ps (dotted line). (b) Trajectories for bipolar Crenel function pulses with durations τ_p=10 ps (full line) and τ_p=5 ps (dotted line). (c) Shape of the effective acoustic pulse fitted from experimental results. (d) Trajectory obtained with the strain pulse displayed in (c).

The reason for the minor effect of the pulse shapes on the final trajectories is that a time dependent change of the anisotropy mostly perturbs the angular momentum alone in contrast to

thermally induced changes of the magnetization. Doing such, the control of the precession essentially occurs via the sequence of pulses and more specifically on the delays between the pulses. Naturally the amplitudes of the pulses also play a role for the magnitude of the precession angles θ and ϕ but not for the temporal sequence for which one obtains for example a suppression or an amplification of the precession. In that sense, our experiments demonstrate an "ideal" case of coherent control using the acoustic pulses. The advantage is twofold. Firstly, the temporal sequence can be controlled with an extreme precision as the pulse sequence comes originally from femtosecond optical pulses. Therefore, even though the acoustic pulses are much longer than the optical pulses, one can control the precession with sub-picosecond resolution. Secondly, the acoustic strain being a weakly dissipative process, one can transport angular momentum over large distances in metallic structures. In fact the largest source of dissipation is the loss at the interface. Therefore, an optimal use of the magneto-acoustic control in multi-layered devices requires adjusting the acoustic impedance at the interfaces.

*email: bigot@unistra.fr

Acknowledgements: The authors acknowledge the financial support of the European Research Council for the Advanced Grant "ATOMAG" ERC-2009-AdG-20090325#247452.

1. Miltat, J., Albuquerque, G. & Thiaville, A. *Spin dynamics in confined magnetic structures I*, (Hillebrands, B. & Ounadjela, K. (ed.)) [1-33] (Topics in Applied Physics, Vol. **83**, Springer-Verlag, Heidelberg, 2002).

2. Kim, J.–W., Vomir, M. & Bigot, J.–Y. Ultrafast magnetoacoustics in nickel films. *Phys. Rev. Lett.* **109**, 166601 (2012).
